# A Comparative Analysis of Hippo Signaling Pathway Components during Murine and Bovine Early Mammalian Embryogenesis

**DOI:** 10.3390/genes12020281

**Published:** 2021-02-16

**Authors:** Jyoti Sharma, Monica Antenos, Pavneesh Madan

**Affiliations:** Department of Biomedical Sciences, University of Guelph, Guelph, ON N1G 2W1, Canada; jsharm02@uoguelph.ca (J.S.); mantenos@uoguelph.ca (M.A.)

**Keywords:** Hippo, blastocyst, MS1/2 (Mammalian Sterile Twenty Like 1 and 2), LATS1/2 (Large Tumor Suppressor 1 and 2), YAP1 (Yes Associated Protein 1), p-YAP1, TAZ (Transcriptional co activator with PDZ-binding motif), TEAD4 (TEA domain transcription factor 4)

## Abstract

The time required for successful blastocyst formation varies among multiple species. The formation of a blastocyst is governed by numerous molecular cell signaling pathways, such as the Hippo signaling pathway. The Hippo signaling pathway is initiated by increased cell–cell contact and via apical polarity proteins (AMOT, PARD6, and NF2) during the period of preimplantation embryogenesis. Cell–cell contact and cell polarity activate (phosphorylates) the core cascade components of the pathway (mammalian sterile twenty like 1 and 2 (MST1/2) and large tumor suppressor 1 and 2 (LATS1/2)), which in turn phosphorylate the downstream effectors of the pathway (YAP1/TAZ). The Hippo pathway remains inactive with YAP1 (Yes Associated protein 1) present inside the nucleus in the trophectoderm (TE) cells (polar blastomeres) of the mouse blastocyst. In the inner cell mass (ICM) cells (apolar blastomeres), the pathway is activated with p-YAP1 present in the cytoplasm. On the contrary, during bovine embryogenesis, p-YAP1 is exclusively present in the nucleus in both TE and ICM cells. Contrary to mouse embryos, transcription co activator with PDZ-binding motif (TAZ) (also known as WWTR1) is also predominantly present in the cytoplasm in all the blastomeres during bovine embryogenesis. This review outlines the major differences in the localization and function of Hippo signaling pathway components of murine and bovine preimplantation embryos, suggesting significant differences in the regulation of this pathway in between the two species. The variance observed in the Hippo signaling pathway between murine and bovine embryos confirms that both of these early embryonic models are quite distinct. Moreover, based on the similarity of the Hippo signaling pathway between bovine and human early embryo development, bovine embryos could be an alternate model for understanding the regulation of the Hippo signaling pathway in human embryos.

## 1. Introduction

### Preimplantation Embryonic Development until Blastocyst Formation

The preimplantation period of development is broadly classified as the time from fertilization to mammalian blastocyst formation. The time required for blastocyst development varies among multiple species, ranging from 4.5 days in mice (*Mus musculus*), to 6–7 days in humans (*Homo sapiens*), to 7–9 days in the cattle (*Bos taurus*) [1,2,3,4]. Blastocyst formation is one of the most critical steps during the period of preimplantation embryogenesis [5].

Successful fertilization of an oocyte with sperm leads to zygote formation [1,2,6]. The zygote then undergoes multiple mitotic divisions, thereby progressing to later stages of embryogenesis. These cell divisions, intrinsically driven by multiple cell signalling pathway and polarity protein complexes, subsequently lead to the process of embryonic lineage specification, leading to the formation of morphologically distinct structures, namely the morula and the blastocyst [1,7,8,9,10].

In the case of mouse embryos, the process of compaction is initiated at the eight-cell stage (approximately day 2.5 post insemination) when the blastomeres compact and adhere to each other to develop into the morula [11,12,13] By comparison, in bovine embryos, compaction is initiated at the 16-cell stage (approximately day 5 post insemination) of preimplantation embryogenesis, thereby leading to the formation of the morula [14]. The nature and type of cells within an embryo change as embryonic development proceeds [15]. Until the two-cell stage of mouse embryogenesis, the blastomeres are totipotent and apolar in nature [16]. However, during the subsequent stages of preimplantation embryogenesis, some of the blastomeres become polarised in nature. By the morula stage, the majority of the mouse blastomeres become pluripotent [16]. Polarisation of the blastomeres can be attributed to a number of apical–basal and basal–lateral polarity protein complexes.

The process of compaction is followed by the formation of blastocoel cavity, known as cavitation, which occurs during the formation of blastocyst [17]. In addition to cavitation, blastocyst formation is characterized by the establishment of two important cell lineages, known as trophectoderm (TE) and inner cell mass (ICM) [18,19]. Multiple classic models, such as “the pre pattering model”, “the inside-outside theory”, and “the cell polarity theory”, can be used to explain the formation of trophectoderm (TE) and inner cell mass (ICM).

According to the “pre patterning theory”, cell fate specification depends on the molecular determinants that are asymmetrically localized in the oocyte during maturation and upon fertilization. These determinants are differentially segregated during the cleavage events following fertilization and the cell fate decision depends on the molecular determinant received by the daughter cell [20,21].

According to the “inside outside theory” postulated by Tarkowski and Wroblewska, the blastomeres present on the outside of a totipotent embryo become the trophectoderm/placenta, whereas the blastomeres present inside the embryo become inner cell mass/fetus [22]. This theory focuses on the position of blastomeres at the eight-cell stage and suggests that the position of the blastomeres dictates the formation of trophectoderm and inner cell mass during the formation of a blastocyst [22].

The “cell polarity theory” postulated by Johnson and Ziomek suggests that, at the eight-cell stage, polarization of the cells is initiated. Polar cells form the outer trophectoderm (TE), whereas the apolar cells become the inner cell mass (ICM) [23].

Each of these three classic models and there combinations have been used to interpret the results related to the process of cell fate specification during blastocyst formation. However, neither of these models can be successfully used to explain the recent advances observed regarding cell fate specification during mouse blastocyst formation [24,25].

Recently, another theory known as the “self-organization theory” postulated by Nodal and Lefty has been shown to explain the new observations made about the process of lineage segregation during mouse blastocyst formation. According to this theory, also known as the “reaction-diffusion pattern mechanism”, the process of cell fate specification in not just dependent upon one single factor, but is dependent on multiple variables such as cell division pattern, cell shape, cell adhesion, and lineage specific gene expression [21,26,27].

Numerous cell signaling pathways, such as the Wnt, Notch, MAPK, and Hippo signaling pathway, play a significant role in cell fate specification and thus are involved in the formation of blastocyst [8,28,29,30,31].

## 2. Hippo Signaling Pathway—Major Components and Their Localization

The Hippo signaling pathway is known to be an important regulator of cell growth and development [32]. The components of this cell signaling pathway can be broadly categorised as follows: upstream regulators, core cascade components, and downstream effectors of the pathway [33].

### 2.1. Upstream Regulators of the Hippo Signaling Pathway

#### 2.1.1. Cell–Cell Contact and Cell Polarity

Various studies conducted in the mouse model demonstrate that cell–cell contact and AMOT (Angiomotin)-NF2-PARD6B mediated cell polarity are the key mediators activating the Hippo signaling pathway [34,35,36]. During early mammalian embryogenesis, cell polarisation and compaction are initiated at the eight-cell stage [37]. This apical–basal and basolateral polarisation of blastomeres, during the post compaction stages, provide clues for lineage segregation during blastocyst formation in preimplantation embryonic development [32,38].

#### 2.1.2. How Do Cell–Cell Contact and Cell Polarity Affect the Hippo Signaling Pathway?

The mouse model is the most widely studied model to illustrate the effects of cell–cell contact on the Hippo signaling pathway components and the subsequent formation of the blastocyst [28]. Inhibition of E-cadherin mediated cell–cell contact has been shown to inactivate the Hippo signaling pathway [28,39]. Inactivation of this cell signaling pathway, as a consequence of E-cadherin mediated cell–cell contact inhibition, interferes with lineage segregation and, in turn, proper blastocyst development during mouse preimplantation embryogenesis [28,39,40].

E-cadherin is expressed during all the post compaction stages of bovine embryogenesis [41]. E-cadherin along with other catenins have been shown to play an essential role in establishing cell–cell contact in early bovine embryogenesis. However, the role of E-cadherin mediated cell–cell contact in the initiation of the Hippo signaling pathway during bovine embryogenesis is yet to be explored.

In addition to cell–cell contact, cell polarity is another important upstream regulator of the Hippo signaling pathway. AMOT (Angiomotin) and Nf2 (neurofibromin type 2) apical polarity proteins are known to be significant regulators of this cell signaling pathway in mouse and bovine embryos [36,42].

AMOT family protein components have been shown to play an important role in the activation of the Hippo signaling pathway during mouse preimplantation embryo development [36]. During mouse blastocyst formation, knockdown of AMOT protein represses the Hippo signaling pathway in the apolar ICM blastomeres, whereas the Hippo signaling pathway still remains active in the polar TE blastomeres [36]. However, in the case of bovine embryogenesis, no effect was observed on the gene expression of YAP1 (yes associated protein 1) and TEAD4 (TEA domain transcription factor 4), the major downstream effectors of the Hippo signalling pathway [42].

Although the presence of AMOT has been established in both mouse and bovine embryos, the role of these cell signaling pathway components in bovine embryogenesis and in the subsequent cell fate specification is still unknown [33,42]. In addition to the AMOT polarity protein, RHOA, a small molecule G protein is known to play a significant role in TE differentiation during bovine blastocyst formation [43]. Specific chemical inhibition of this protein molecule inhibits YAP1, thereby playing an important role in TE differentiation during bovine blastocyst formation [43].

**Figure 1:**
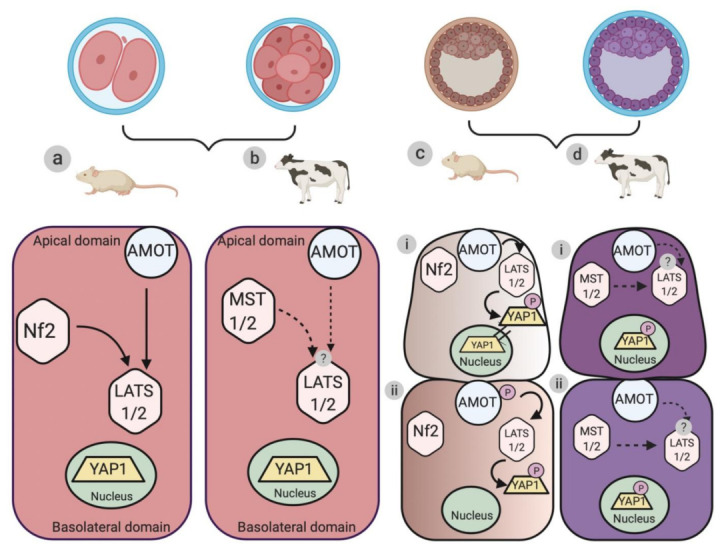
Schematic illustration of the differences in the localization of the upstream regulators and the core cascade components of the Hippo signaling pathway during murine and bovine embryogenesis. (**a**) Organization hierarchy of the upstream regulators (AMOT and Nf2) and core cascade components (large tumor suppressor 1 and 2 (LATS1/2) protein kinase) of the Hippo signaling pathway during pre-compaction stages (two-cell to eight-cell stages) of mouse embryogenesis. Arrows represent the direction of activation of the protein kinase components. (**b**) Protein localization of the upstream regulators (AMOT and Nf2) and the core cascade components (mammalian sterile twenty like 1 and 2 (MST1/2) and large tumor suppressor 1 and 2 (LATS1/2)) of the Hippo signaling pathway during the pre-compaction stages of bovine embryogenesis. Dotted arrows represent a potential link in the activation of the protein kinase components. (**c**,**d**) Localization of AMOT, Nf2, MST1/2, and LATS1/2 in TE (trophectoderm) and ICM (inner cell mass) during blastocyst formation in murine and bovine models, respectively. (**c**-i) The Hippo signaling pathway is inactive (Hippo “Off”) in the outer polar TE cells, where AMOT and Nf2 cause the nuclear retention of YAP1. (**c**-ii) The Hippo signaling pathway is active (Hippo “On”) in the apolar ICM cells, where AMOT and Nf2 cause the phosphorylation of LATS1/2 and the subsequent cytoplasmic retention of p-YAP1. (**d**-i and ii) Protein localization of upstream regulators (AMOT) and core cascade components (MST1/2 and LATS1/2) of Hippo signaling pathway components in TE and ICM during bovine blastocyst formation. Dotted lines represent the proposed mechanism of functioning of the Hippo signaling pathway during (i) TE and (ii) ICM formation.

Nf2 is another membrane-bound protein known to play a significant role in embryonic cell fate specification [34]. Inhibition of Nf2 causes differences in YAP1 localization, thereby aiding in the segregation of TE and ICM during mouse blastocyst formation [34,36]. This suggests that Nf2 plays a vital role as an upstream regulator of the Hippo signaling pathway in mouse embryogenesis (Figure 1).

### 2.2. Core Components of the Hippo Signaling Pathway—MST and LATS Protein Kinase

#### 2.2.1. MST Protein Kinase

The core components of the Hippo signaling pathway include MST1/2 (mammalian sterile twenty like 1 and 2) and LATS 1/2 (large tumor suppressor 1 and 2) [44].

MST protein kinase was first discovered in *Drosophila melanogaster* (fruit fly) and termed as ‘*Hpo*’, as deletion of this gene led to tissue overgrowth, thereby gaining the phenotype of a hippopotamus [45,46]. MST protein kinase plays a significant role in inhibiting cell proliferation and promoting apoptosis during mouse embryogenesis [47,48]. Studies of mouse embryonic stem cells and embryos suggest that MST1 and 2 can functionally compensate for each other [47,49]. *Mst* 1 or 2 single knockout mice were fertile and showed normal embryonic development with no significant developmental or immunological defects [47]. In contrast, *Mst1/2−/−* double knockout mice died in utero with severe developmental defects, after 8.5 days of embryonic development [47]. 

Similarities have been observed in the localization of MST1 and 2 in bovine and mouse embryos [47,49]. In both species, MST1 and 2 are localised in the cytoplasm, suggesting a similar role of these components in both mouse and bovine early embryo development (Figure 1).

#### 2.2.2. LATS Protein Kinase

In addition to MST protein kinase, the core cascade of the Hippo signaling pathway consists of LATS1 and 2 [44,50,51]. LATS protein kinase plays a significant role in cell fate specification and the subsequent formation of the inner cell mass during mouse blastocyst development [28,51]. LATS protein kinase regulates the nuclear-cytoplasmic shutting of YAP1 and TAZ (transcription co activator with PDZ-binding motif). During early mouse embryonic development, LATS protein kinase is known to phosphorylate YAP1 and retain the protein in the cytoplasm of the inner cells of the blastocyst [28,51]. Knockdown of LATS1/2 protein kinase, at the zygote stage, increases nuclear YAP localisation in the inner cells during preimplantation mouse embryogenesis [28]. Nuclear localization of YAP1 causes inactivation of the Hippo signaling pathway in the inner cells of the mouse blastocyst, whereas in the outside cells, LATS protein kinase causes YAP1 phosphorylation, leading to increased cytoplasmic retention of p-YAP in the outside cells.

Even though the localization and role of LATS1 and 2 are well understood in early mouse embryos, no such information is available regarding preimplantation bovine embryogenesis (Figure 1). Future experiments should focus on inhibiting LATS1/2 to establish the mechanism of action of the Hippo pathway during bovine embryogenesis.

#### 2.2.3. How Do the Core Cascade Components of the Hippo Signaling Pathway Affect Mammalian Embryo Development?

LATS protein kinase has been suggested to be the inter-mediatory link relaying signals between E-cadherin mediated cell–cell contact and YAP1. During mouse preimplantation embryogenesis, LATS protein kinase acts as a key regulator in the transmission of E-cadherin mediated cell–cell contact cues to the activation of the Hippo signaling pathway [52]. During mouse blastocyst formation, LATS mediated Hippo signaling is inactive in the outer cells (TE), whereas this cell signaling is active in inner cells (ICM) owing to phosphorylation of LATS (p-LATS), thereby leading to activation of the Hippo signaling pathway [36,52] (Figure 1).

### 2.3. Downstream Effectors of Hippo Signaling Pathway

#### 2.3.1. YAP1 and TAZ

Recently, various studies have suggested that YAP1 and TAZ (or WWTR1) are key regulators of numerous other cell signaling pathways in addition to the Hippo signaling pathway [53], and thus could be an important conduit for the cross talk between various other signaling pathways during early embryogenesis.

In the mouse model, translocation of Hippo signaling pathway co-activators, YAP and TAZ, between the nucleus and cytoplasm cause the activation or inactivation of this signaling pathway [28,51,53]. When the cell signaling pathway is active (ON), YAP and TAZ are present in the cytoplasm in their phosphorylated forms (p-YAP and p-TAZ), whereas when this cell signaling pathway is inactive (OFF), YAP/TAZ enter the nucleus and cause activation of TEAD family transcription factors. During preimplantation mouse embryogenesis, YAP and TAZ are present in the cytoplasm during the pre-compaction stages, but are localized in the nucleus during the post compaction stages.

Localization of the co-activators of the Hippo signaling pathway is different between mouse and bovine embryos [28,33,44,54]. During mouse embryogenesis, p-YAP1 is present in the cytoplasm at stages of preimplantation embryo development. During bovine embryogenesis, TAZ is present in the cytoplasm during the pre-compaction stages; after the eight-cell stage, however, TAZ is localised in the nucleus in some of the blastomeres [33].

**Figure 2:**
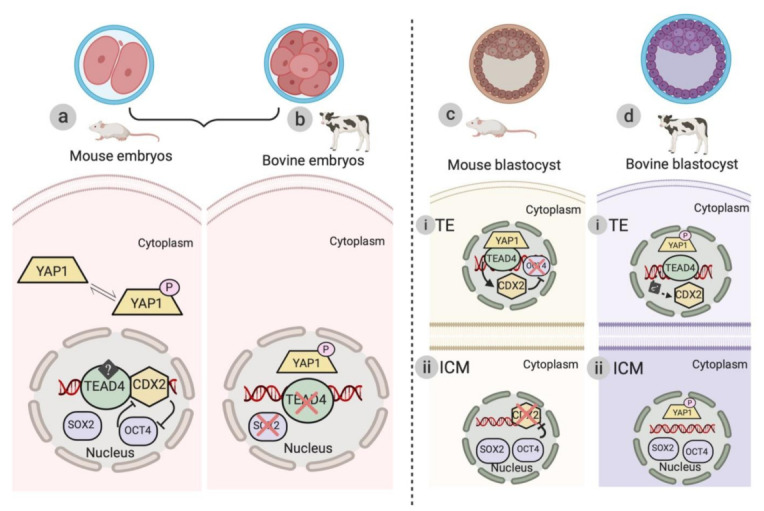
Schematic illustration of the differences in the localization of downstream effectors of the Hippo signaling pathway during murine and bovine embryogenesis. (**a**) Mechanism of activation of TEAD4 and other transcription factors (CDX2, SOX, and OCT4) of the Hippo signaling pathway during pre-compaction stages (from two-cell to eight-cell stage) of mouse preimplantation embryogenesis. The arrows represent the inhibitory mechanism between CDX2 and OCT4 in the murine model. (**b**) Protein localization of downstream effectors (p-YAP1, TEAD4, and SOX2) of the Hippo signaling pathway. p-YAP1 is localized in the nucleus and untraceable amounts of TEAD4 and SOX2 are detected during the pre-compaction stages of early bovine embryogenesis. (**c** and **d**) Localization of YAP1/p-YAP1 and TEAD4 transcription factors (CDX2, SOX2, and OCT4) in TE (trophectoderm) and ICM (inner cell mass) during blastocyst formation in murine and bovine models, respectively. (**c**-i) In the outer polar TE cells (Hippo “Off”), YAP1 in collaboration with TEAD4 causes the transcription of CDX2. OCT4 expression is thus inhibited by CDX2, aiding in the process of TE differentiation. (**c**-ii) In the apolar ICM cells, the Hippo signaling pathway is active (Hippo “On”) and SOX2 and OCT4 are exclusively present in the nucleus. In the ICM, OCT4 supresses the expression of CDX2 and facilitates the formation of ICM. (**d**-i and ii) Protein localization of downstream effectors (p-YAP1) and TEAD4 transcription factors (CDX2, SOX2, and OCT4) of the Hippo signaling pathway in TE and ICM during bovine blastocyst formation. The dotted line represents the proposed mechanism of TEAD4 and CDX2 interaction during bovine TE differentiation.

In addition to TAZ, YAP1/p-YAP1 is another important downstream regulator of the Hippo signaling pathway. Similar to mouse embryos, YAP1 is localized in the nucleus during pre- and post-compaction stages of development (Negrón-Pérez and Hansen 2017). By contrast, localization of p-YAP has been shown to be distinct in bovine embryos in comparison with mouse embryos [33]. In the pre-compacted bovine embryos, p-YAP has been shown to be present in both the nucleus and cytoplasm, whereas in the post compaction stages of bovine preimplantation, embryogenesis p-YAP is predominantly present only inside the nucleus [33] (Figure 2). This distinct localization of the downstream effectors of the Hippo signaling pathway in early bovine embryogenesis suggests that this pathway is differentially activated in bovine embryos as compared with mouse embryos. Further chemical inhibition and/or siRNA knockdown studies are required to validate the role of the Hippo signaling pathway components in bovine embryos.

#### 2.3.2. How Do the Downstream Effectors of the Hippo Signaling Pathway Affect Mammalian Preimplantation Embryo Development?

YAP1 and TAZ are the major downstream effectors of the Hippo signaling pathway. Nucleo-cytoplasmic localization of these co-activators affects cell growth and proliferation. Recently, various pharmacological inhibitors, such as Statins and Verteporfin, as well as epigenetic alterations, such as GapmeR antisense oligo nucleotide treatment and siRNA knockdown, have been performed to inhibit Hippo signaling pathway co-activators, YAP1 and TAZ, in multiple human cell lines and mammalian embryos [42,55,56]. Chemical inhibition, such as Statin treatment, has been shown to diminish the size of the blastocoel cavity and decrease the nuclear expression of YAP1 in mouse embryos; however, no such information is available in the bovine model. Recent reports suggest differences in the localization of the downstream effectors (p-YAP1 and TAZ) of the Hippo signaling pathway components in bovine embryos as compared with the established hierarchy of the signaling pathway in the mouse embryos. Further inhibition studies are required to investigate the role of nuclear p-YAP1 [9,35,36]. These inhibition studies will demonstrate the role of these cell signaling pathway components in cell fate specification and the subsequent differentiation of TE and ICM.

#### 2.3.3. TEAD Family as the Downstream Effectors of YAP1 and TAZ

TEAD4 induces the transcription of CDX2 (caudal type homeobox 2), SOX2 (SRY-related HMG box 2), and OCT4 (octamer binding transcription factor 4, also known as POU5F1), which have been established as the important biomarkers for TE and ICM specification [28,54,57]. During preimplantation mouse embryogenesis, CDX2 has been established as the significant maker for the TE cells, whereas SOX2 and OCT4 are important ICM markers [8,9,54,57,58].

During mouse embryogenesis, interaction of TEAD family transcription factors (especially TEAD4) with YAP1 plays an essential role in the formation of TE [28,59]. TEAD4 transcripts are present during all stages of mouse embryogenesis; however, the nuclear localization of TEAD4 is initiated at the 8–16-cell stage. When present inside the nucleus, TEAD4 causes the transcription of CDX2. Inhibition of TEAD4 decreases the expression of CDX2; however, no such effect was observed on the ICM transcription factors [59]. Another study further validated these results by elucidating that the overexpression of TEAD4 has been shown to cause a parallel increase in the expression of CDX2. This finding suggests that TEAD4 is an important regulator of TE formation [28]. SOX2 is expressed in the nucleus during all the stages of mouse preimplantation embryo development [60]. During the formation of a blastocyst, SOX2 is exclusively present in the nucleus of apolar ICM cells [60]. SOX2 is the initial most pluripotency marker known to be expressed in the inner cells of the mouse blastocyst [61,62]. Similar to SOX2, another ICM marker, OCT4, has been shown to be universally present during all the stages of mouse embryogenesis; however, the expression peaks at the 16-cell stage and the nuclear presence of OCT4 are restricted to the ICM during blastocyst formation [63]. Another study suggests that the role and expression of OCT4 are dependent on the ability of OCT4 to form a complex with CXD2, where CDX2 is responsible for the inhibition of OCT4 expression in the TE cells during the formation of blastocyst [64] (Figure 2).

In the case of bovine embryos, TEAD4 did not significantly affect the gene expression of CDX2 or other TE/ICM transcription factors such as OCT4 and SOX2 [65]. In contrast to mouse embryos, where SOX2 expression was initiated at the two-cell stage, the protein expression of SOX2 was initiated at the 16-cell stage of embryogenesis [66]. These conflicting reports suggest potential differences in the mechanism of TE and ICM formation as well as differentiation during early mouse and bovine embryonic development [59,65] (Figure 2).

## 3. Unanswered Questions About the Hippo Signaling Pathway during Embryogenesis

Is the activation of the Hippo signaling pathway dependent on cell–cell contact or cell polarity in early bovine embryos? Although the presence of the AMOT polarity protein has been established during both mouse and bovine embryogenesis, further studies are required to reveal the role of this polarity protein in the activation of the Hippo signaling pathway during bovine blastocyst formation [42]. A second question remains to be answered about the role of MST1 and 2 during bovine embryogenesis. The localization of MST1 and 2 has been established to be the same as mouse embryos, however, the function of this protein kinase during bovine embryogenesis still remains unknown (Sharma and Madan, 2019). A third question pertains to the core cascade components (LATS1/2) of the Hippo signaling pathway. Is the function of LATS1/2 during bovine embryogenesis dependent on the signal received from MST1/2 protein kinase or does it act independently of MST kinase and receive direct signals from the AMOT polarity protein, in a manner similar to that established during mouse embryogenesis [28,51]? This information will fill the existing gaps in our knowledge pertaining to the mechanism of the Hippo signaling pathway activation during early bovine embryo development and will shed more light on the process of blastocyst formation. Lastly, why is p-YAP1 translocated to the nucleus during all the stages of early bovine embryogenesis [33]? Further studies about p-YAP1 and other downstream effectors (YAP1, TAZ, p-TAZ, TEAD4, CDX2, SOX2, and OCT4) of the Hippo signaling pathway will help us better understand the process of cell fate specification and the concept of lineage segregation during the formation of a blastocyst.

## 4. Conclusions

The localization of Hippo signaling pathway components is significantly different between bovine and mouse embryogenesis, suggesting the Hippo signaling pathway is differentially regulated in bovine embryos as compared with mouse embryos [33,51,59,65] (Figure 2). The nuclear localization of p-YAP1 might be due to some transporter proteins working in association with nuclear pore complexes present on the nuclear membrane [67]. Further studies need to be performed to investigate the involvement and/or regulation of these transporter proteins in the transport of p-YAP1 to and from the nucleus and cytoplasm in bovine embryos. These studies will shed more light on the organization hierarchy of Hippo signaling pathway components in the bovine model and will further establish the role of this cell signaling pathway in cell fate specification during the formation of the bovine blastocyst. Understanding the biology of bovine blastocyst formation will also enhance our knowledge about the mechanism of human blastocyst development, as multiple reports suggest that the regulation of human blastocyst formation is more similar to that of bovine as compared with the mouse model [68,69]. The study of Hippo signaling pathway components in various species will help us better understand the biology of mammalian blastocyst formation. Further studies pertaining to the mechanism of activation and localization of Hippo signaling pathway components will improve our knowledge about the processes of cell fate specification and lineage segregation during the formation of a blastocyst.

## Data Availability

The data presented in this study is openly available.
